# Pneumococcal Pneumonia and the Risk of Stroke: A Population-Based Follow-Up Study

**DOI:** 10.1371/journal.pone.0051452

**Published:** 2012-12-12

**Authors:** Li-Fu Chen, Hsin-Pai Chen, Yung-Sung Huang, Kuang-Yung Huang, Pesus Chou, Ching-Chih Lee

**Affiliations:** 1 Department of Emergency Medicine, National Yang-Ming University Hospital, I-Lan, Taiwan; 2 Department of Internal Medicine, National Yang-Ming University Hospital, I-Lan, Taiwan; 3 Division of Neurology, Department of Internal Medicine, Buddhist Dalin Tzu Chi General Hospital, Chiayi, Taiwan; 4 Division of Allergy, Immunology, and Rheumatology, Department of Internal Medicine, Buddhist Dalin Tzu Chi General Hospital, Chiayi, Taiwan; 5 Department of Otolaryngology, Buddhist Dalin Tzu Chi General Hospital, Chiayi, Taiwan; 6 Cancer Center, Buddhist Dalin Tzu Chi General Hospital, Chiayi, Taiwan; 7 Center for Clinical Epidemiology and Biostatistics, Buddhist Dalin Tzu Chi General Hospital, Chiayi, Taiwan; 8 School of Medicine, Tzu Chi University, Hualian, Taiwan; 9 Community Medicine Research Center and Institute of Public Health, National Yang-Ming University, Taipei, Taiwan; Albert Einstein College of Medicine, United States of America

## Abstract

**Background:**

To investigate the risk of developing stroke in patients hospitalized following a diagnosis of pneumococcal pneumonia.

**Methods:**

The study cohorts comprised of patients hospitalized with a principal diagnosis of pneumococcal pneumonia (*n*  = 745), with a random sampling of control individuals in 2004 (*n*  = 1490). The Cox proportional hazard model was used to compare the stroke-free survival rate between the cohorts after adjusting for possible confounding and risk factors for a two-year follow up. Instrumental variable analysis (IVA) was used to address potential biases associated with measured and unmeasured confounding variables.

**Results:**

Of the 153 patients with stroke, 80 (10.7%) were from the pneumococcal pneumonia cohort, and 73 (4.9%) were from the control group. The risk of stroke was 3.65 times higher (95% confidence interval, 2.25–5.90; P<0.001) in patients with pneumococcal pneumonia after adjusting for patient characteristics, co-morbidities, geographic region, urbanization level of residence, and socioeconomic status during the first year. IVA showed an additional 14% risk of stroke for pneumococcal pneumonia patients (odds ratio = 1.14; 95% CI, 1.02–1.26, P = 0.032).

**Conclusions:**

Patients with pneumococcal pneumonia carry an increased risk for stroke than the general population. Further studies are warranted for developing better diagnostic and follow-up strategies for patients with increased risk.

## Introduction

Atherosclerosis is closely related to infectious diseases [1], [2], [3]. In addition to the variety of infectious agents implicated in atherogenesis [4], acute systemic infections also create a condition that predisposes patients to intravascular thrombosis through the production of inflammatory cytokines, which alter endothelial functions and cause atheromas to become unstable [5]. Systemic respiratory tract infection has been shown to be closely related to subsequent vascular thrombotic events. The risks of myocardial infarction and stroke were 4.95 and 3.19 times higher, respectively, in patients with acute systemic respiratory infection than in those without, whereas these risks were higher–but not to the same extent as in the former patient group–in patients with urinary tract infection than in those without [6].

Community-acquired pneumonia (CAP) produces frequently high levels of circulating proinflammatory cytokines and is associated with significant morbidity and mortality [7]. Because systemic inflammation is closely related to intravascular thrombotic events, a reasonable presumption is that patients suffering from severe CAP bear a high risk for myocardial infarction and stroke [8].

The relationship between arterial thrombotic events and specific causative pathogens of CAP has rarely been analyzed. Of all pathogens, infection by *Streptococcus pneumoniae* is severe and most commonly results in hospitalization. In cerebrovascular events, a direct link between pneumococcal pneumonia and stroke has not yet been demonstrated. To address this, we sought to determine the incidence of stroke in patients with pneumococcal pneumonia identified through the National Health Insurance Research Database (NHIRD) in Taiwan. This nationwide, population-based data set allows for tracing of the medical history of all citizens and provides a unique opportunity to compare stroke risk in patients with pneumococcal pneumonia patients without such risk.

## Methods

### Study Population

This study used the 2004 to 2007 NHIRD published by Taiwan’s National Health Research Institutes. The NHIRD covered medical benefit claims for approximately 98% of the population and contained a registry of board-certified physicians and contracted medical facilities. Because the data consists of de-identified secondary data released to the public for research, this study was exempted from full review by the Institutional Review Board.

The study design featured a study cohort and comparison cohort. The study cohort included all patients who were hospitalized with a principal diagnosis of pneumococcal pneumonia (ICD-9-CM codes 481) in 2004.

The control cohort included the general population from the “Longitudinal Health Insurance Dataset (LHID)” released by the Taiwan Nation Health Research Institute for the period of 2004 to 2006. This dataset consists of 1,000,000 subjects randomly selected from the entire set of enrollees. There were no significant differences in age, gender, or health care costs between the sample group and all enrollees, as reported by the Taiwan National Health Research Institute (www. nhri.org.tw).

Patients with any type of stroke (ICD-9-CM 430–438) diagnosed before or during the ambulatory visit or pneumococcal pneumonia (ICD-9-CM codes 481) in 2004 were excluded from both cohorts.

We identified 432,311 individuals without pneumococcal pneumonia from the LHID, which represented the general population, and 745 patients with pneumococcal pneumonia, aged 18 years or above. Because of significant difference in the mean age between the two groups, the control cohort criteria were further refined by randomly selecting two age-matched appendectomy patients (i.e., aged less than 45, 45 to 64, 65 to 74, and 75 years or above) for every pneumonia patient. Each patient was tracked from the first hospitalization (for the pneumonia group) or first ambulatory visit (for the control group) for a two-year period using administrative data to identify all patients who developed any type of stroke (ICD-9-CM codes 430–438). These patients were then linked to the administrative data covering the 2004 to 2006 period to determine the stroke-free survival time, with cases censored for patients who drew back guarantees from the National Health Insurance Program or were still robust without defined events at end of follow-up. The independent variables were gender and comorbid disorders.

Details on medical comorbidities, including hypertension, diabetes, coronary artery disease, hyperlipidemia, atrial fibrillation, chronic renal disease, obesity, and peripheral arterial disease, were extracted from the claims data at the time of index discharge. These conditions were then associated with vascular events.

### Statistical Analysis

The SAS statistical package (version 9.2; SAS Institute, Inc., Cary, NC, USA) and SPSS (version 15; SPSS Inc., Chicago, IL, USA) were used for data analysis. The cumulative risk of stroke was estimated as a function of time from initial treatment. Pearson’s chi-square test was used for categorical variables in the two cohorts, whereas the two-year stroke-free survival rate was estimated using the Kaplan–Meier method. The Cox proportional hazard regression model was used to calculate the stroke risk in patients with pneumococcal pneumonia versus controls after adjustments for variables. *P<*0.05 was considered statistically significant in the regression models. The positive predictive rate for pneumococcal pneumonia with ICD-9 481 was 56.8% to 63.9% for definite and probable pneumococcal pneumonia cases [9]. So we did a sensitivity analysis under a positive predictive rate of 50% for pneumococcal pneumonia in our series.

Instrumental variable analysis (IVA) from Rubin Causal Model was used to account for both the measured and unmeasured confounding factors [10]. The instrumental variable was constructed by first calculating the proportion of pneumococcal pneumonia in each residence area. The algorithm produced 144 residence areas. The residence area was divided into quintiles according to the proportion of pneumococcal pneumonia. During preliminary analysis, the characteristics of the top quintile with the highest proportion of pneumococcal pneumonia residence area were quite different from the others, and it was eliminated from the IVA. An instrumental variable must be associated with outcomes through its correlation with disease status (pneumococcal pneumonia) and not through other covariates. The average effect of pneumococcal pneumonia was as follows:

IV estimate = (Stroke rate _Hi_-Stroke rate _Lo_)/(Pr(pneumonia | Hi)-Pr(pneumonia | Lo) in which “Hi” indicates an area with a high rate of pneumococcal pneumonia and “Lo” indicates an area of low rate of pneumococcal pneumonia.

We verified this assumption by comparing the baseline characteristics, including age at diagnosis, gender, and comorbidities. Two-stage least squares were used to estimate the effect of pneumococcal pneumonia on stroke risk using instrumental variables.

## Results

Distributions of demographic characteristics and selected comorbidities for the two cohorts are shown in Table 1. Compared to the controls, patients with pneumococcal pneumonia were more likely to be men and those with underlying diseases, including hypertension, diabetes, coronary heart disease, atrial fibrillation, and chronic renal disease.

**Table 1:** Demographic characteristics and comorbidities of the pneumococcal pneumonia and control groups in Taiwan, 2004 (n = 2235).

Variable	Pneumococcal pneumonia group (n = 745)		Control group (n = 1490)		P-value
	n	(%)	n	(%)	
Gender					<0.001
Male	496	(67)	742	(50)	
Female	249	(33)	748	(50)	
Age, years					1.000
18–44	132	(18)	264	(18)	
45–64	165	(22)	330	(22)	
65–74	172	(23)	344	(23)	
≧75	276	(37)	552	(37)	
Hypertension					<0.001
Yes	122	(16)	90	(6)	
No	623	(84)	1400	(94)	
Diabetes					<0.001
Yes	129	(17)	38	(3)	
No	616	(83)	1452	(97)	
Coronary heart disease					<0.001
Yes	60	(8)	33	(2)	
No	685	(92)	1457	(98)	
Hyperlipidemia					0.430
Yes	11	(2)	29	(2)	
No	734	(98)	1461	(98)	
Atrial fibrillation					<0.001
Yes	19	(3)	5	(0.3)	
No	726	(97)	1485	(99.7)	
Chronic renal disease					0.001
Yes	31	(4)	27	(2)	
No	714	(96)	1463	(98)	
Obesity					0.157
Yes	0	(0)	4	(0.3)	
No	745	(100)	1486	(99.7)	
Peripheral arterial disease					0.016
Yes	1	(0.1)	16	(1)	
No	744	(99.9)	1474	(99)	

**Figure 1:**
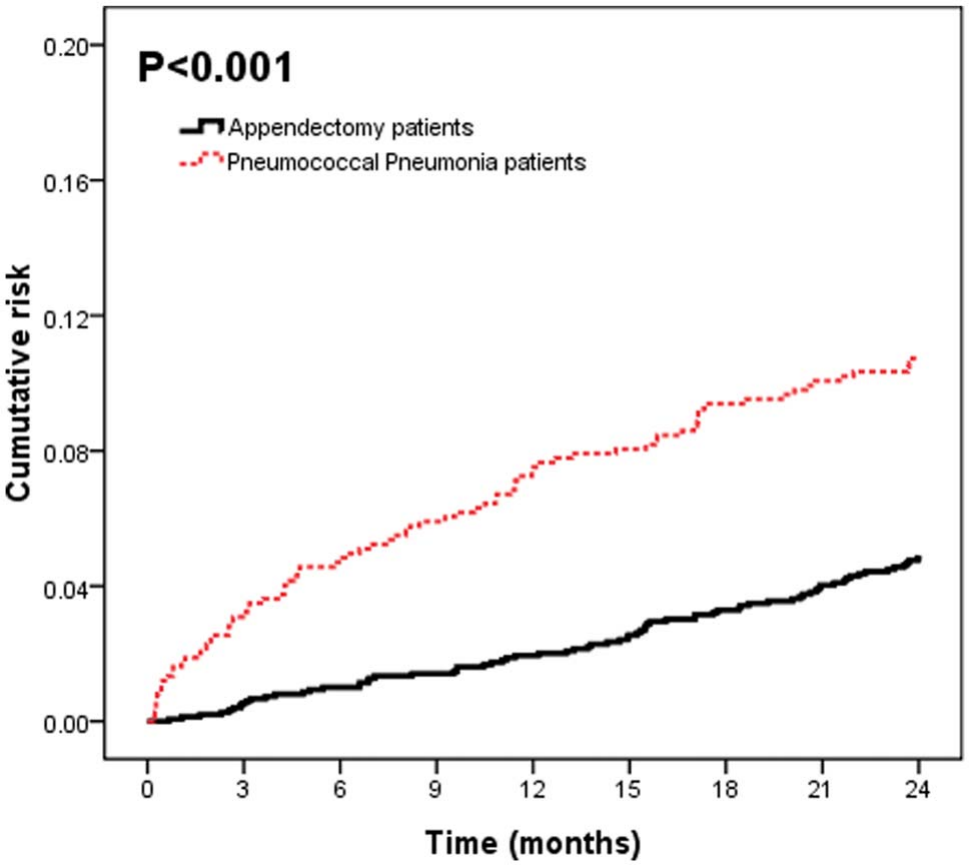
Cumulative risk of stroke for the pneumococcal pneumonia patients and the controls.

At the end of follow-up, 153 patients had strokes, with 80 (11%) from the pneumococcal pneumonia group and 73 (5%) from the control group (Fig. 1). The cumulative risk of stroke was 3.2% (95% confidence interval [CI], 2.0–4.4) for the three-month follow-up, 7.5% (95% CI, 5.5–9.5) for the one-year follow-up, and 10.7% (95% CI, 8.5–12.9) at the end of follow-up (Table 2). Table 3 shows, in a model over time, the estimated of hazard ratios of pneumococcal pneumonia were less than 1, which pointed out that the effects of pneumococcal pneumonia are much stronger at the beginning of the follow-up period and weaker after long-term follow-up. The observational period was further separated into the first year and the second year. Table 4 revealed that the effect of pneumococcal pneumonia for stroke was much stronger in the first year (HR = 3.65; 95% CI, 2.25–5.90).

**Table 2:** Cumulative risk of stroke events in patients with pneumococcal pneumonia and those without.

Variable	3 months		1-year		2-year	
	Events (%)	Risk (%) (95%CI)	Events (%)	Risk (%) (95%CI)	Events (%)	Risk (%) (95%CI)
Pneumococcal pneumonia group (n = 745)	24 (3.2)	3.2 (2.0–4.4)	56 (7.5)	7.5 (5.5–9.5)	80 (10.7)	10.7 (8.5–12.9)
Control group (n = 1490)	8 (0.5)	0.5 (0.49–0.5)	29 (1.9)	2.3 (2.2–3.1)	73 (4.9)	4.9 (3.7–6.1)

**Table 3:** Hazard ratios of stroke events among 2235 patients.

Variable	Model without time interactions			Model with time interactions		
	HR	95%CI	P value	HR	95%CI	P value
Pneumococcal pneumonia	1.95	(1.4–2.8)	<0.001	0.78	(0.5–1.2)	0.295
Male	1.29	(0.9–1.8)	0.143	1.47	(1.0–2.1)	0.031
Age, years	1.05	(1.0–1.1)	<0.001	1.04	(1.0–1.1)	<0.001
Hypertension	1.28	(0.8–2.0)	0.273	1.03	(0.75–1.6)	0.897
Diabetes	1.34	(0.8–2.2)	0.235	1.22	(0.7–2.1)	0.473
Coronary heart disease	0.46	(0.2–1.1)	0.069	0.88	(0.4–2.1)	0.768
Hyperlipidemia	1.49	(0.57–4.7)	0.496	1.99	(0.6–6.3)	0.243
Atrial fibrillation	2.09	(0.9–4.8)	0.081	3.35	(1.4–7.96)	0.005
Chronic renal disease	1.90	(1.0–3.5)	0.038	1.01	(0.5–1.9)	0.986
Obesity	–	–	–	–	–	–
Peripheral arterial disease	3.72	(1.4–10.2)	0.011	0.99	(0.3–3.0)	0.981
Pneumococcal pneumonia*log(time)	–	–	–	0.01	(0.01–0.02)	<0.001

Abbreviation: HR, hazard ratio; 95% CI, 95% confidence interval.

**Table 4:** Hazard ratio of stroke among the pneumococcal pneumonia patients and the control group stratified by time; 85 stroke events in the first year of follow-up and 68 events in the second year of follow-up.

Variable	Event in the 1^st^ year				Event in the 2^nd^ year			
	No. of events	HR	95%CI	P value	No. of events	HR	95%CI	P value
Pneumococcal pneumoniagroup (n = 745)	56	3.65	(2.25–5.90)	<0.001	24	0.91	(0.53–1.59)	0.750
Control group(n = 1490)	29	1	–	–	44	1	–	–

Abbreviation: HR, hazard ratio; 95% CI, 95% confidence interval.

We explored whether the association between pneumococcal pneumonia and stroke could be due to underlying diseases such as hypertension, diabetes, coronary artery disease, hyperlipidemia, atrial fibrillation, chronic renal disease, obesity, and peripheral arterial disease (Table 5). Pneumococcal pneumonia remained an independent risk factor for stroke irrespective of a prior history of other comorbidities.

**Table 5:** Adjusted hazard ratios of stroke events among the individuals with pneumococcal pneumonia and those without pneumococcal pneumonia stratified by comorbidities for the first year of follow-up.

Variable	No. of stroke events, n (%)	Age and gender adjusted HR (95% CI)	Multivariate adjusted HR (95% CI)
***With other comorbidities*** * ***(n = 1764)***			
Pneumococcal pneumonia group (n = 475)	29 (6.1)	3.55(1.45–8.68)	5.00(1.78–14.07)
Control group(n = 1289)	23 (1.8)	1	1
			
***Without other comorbidities*** * ***(n = 471)***			
Pneumococcal pneumonia group (n = 270)	27 (10)	3.23(1.86–5.62)	3.23(1.86–5.62)
Control group (n = 201)	6 (3)	1	1

Abbreviation: HR, hazard ratio; 95% CI, 95% confidence interval.

* Comorbidities included hypertension, diabetes, coronary artery disease, hyperlipidemia, atrial fibrillation, chronic renal disease, obesity, and peripheral arterial disease.

For sensitivity analysis, 50% of 745 pneumococcal pneumonia patients were randomly assigned as the “real case group”. The other 50% patients were grouped with 1,490 individuals as the “real control group”. Appendix S1 reveals that pneumococcal pneumonia remained a risk factor for stroke during the first year of follow-up.

Among the instrumental variable analysis, there were no statistical differences between the patients’ characteristics in high-pneumococcal pneumonia and low-pneumococcal pneumonia areas (Table 6). For the first year of follow-up, 54 patients had stroke events, 34 (4.1%) were patients in high-pneumococcal pneumonia areas and 20 (2.4%) were patients in low-pneumococcal pneumonia areas. When instrumental variable analysis was used alongside the two-stage least squares technique, pneumococcal pneumonia was statistically associated with the infection event (odds ratio [OR], 1.14; 95% CI, 1.02–1.26; P = 0.032) for the first year of follow-up (Table 7). There was no statistical association between the pneumococcal pneumonia and stroke for the two-year follow–up period.

**Table 6:** Characteristics of individuals in high-pneumococcal pneumonia and low-pneumococcal pneumonia areas (n = 1640).

	High(n = 820)		Low(n = 820)		P-value
	N	(%)	n	(%)	
Age,yr (Mean±SD)	63±19		64±19		0.426
Male	458	(56)	436	(53)	0.275
Hypertension	68	(8)	68	(8)	1
Diabetes	61	(7)	46	(6)	0.134
Coronary heart disease	36	(4)	21	(3)	0.043
Hyperlipidemia	10	(1)	20	(2)	0.065
Atrial fibrillation	7	(1)	4	(1)	0.364
Chronic renal disease	16	(2)	20	(2)	0.500
Obesity	2	(0.2)	1	(0.1)	0.563
Peripheral arterial disease	4	(1)	8	(1)	0.246

**Table 7:** Marginal effect of pneumococcal pneumonia on stroke event using instrumental variable analysis for one-year follow up (n = 1640).

	Odds ratio	(95%CI)	P-value
Pneumococcal pneumonia	1.14	(1.02–1.26)	0.032
Age, yr	1.00	(1.001–1.002)	<0.001
Male	1.00	(0.98–1.02)	0.979
Hypertension	0.98	(0.94–1.02)	0.303
Diabetes	0.97	(0.91–1.03)	0.291
Coronary heart disease	0.93	(0.88–0.98)	0.008
Hyperlipidemia	1.01	(0.95–1.08)	0.687
Atrial fibrillation	0.91	(0.80–1.03)	0.123
Chronic renal disease	1.11	(1.04–1.17)	0.002
Obesity	1.02	(0.81–1.22)	0.879
Peripheral arterial disease	1.09	(0.98–1.19)	0.138

Abbreviation: 95% CI, 95% confidence interval.

## Discussion

In this study, we clearly demonstrated that there is an increase in the risk of stroke, which lasts at least one year after acute pneumonia caused by *S. pneumoniae*. Pulmonary infection by *S. pneumoniae* seems to play a role in “triggering” stroke in patients with a high risk of stroke. This deleterious influence did not wane until one year after resolution of pneumonia.

The strengths of our study are that it is a population-based study (n = 2,235) in Taiwan, the study experienced almost complete follow-up of with infectious events across the entire study population, and experienced regular monitoring of the accuracy of diagnosis and treatment by the National Health Insurance Bureau of Taiwan. Besides traditional Cox regression analysis, we further performed instrumental variable analysis. Using IVA to control both the measured and unmeasured confounding factors, there was an additional 13% risk of stroke for pneumococcal pneumonia patients. Severity of comorbidities, tobacco use, dietary habit and a few social factors, such as employment and patient preferences, are difficult to monitor accurately from the dataset. All these unmeasured factors could produce significant bias using traditional approaches. IVA was chosen in order to simulate the randomization process with the observational study. The instrumental variable was verified by comparing the baseline characteristics, which found that these factors were similar between the high-pneumonia areas and low-pneumonia areas. Instrumental variable analysis produced estimates that were less bias.

The results of the present study are in agreement with those previously reported by Smeeth et al [6], which showed acute lower respiratory tract infections were associated with a transient increase in the risk of a vascular event up to 90 days. Several novel points were found in this study. First, a longer post-infection period for stroke risk was observed in the present study, which focuses on the influence of a specific disease-pneumococcal pneumonia, rather than a more generalized diagnosis of acute lower respiratory tract infection. This result suggested that the risk for infection-associated stroke might be different depending on the type of infection or the offending pathogen. Community-acquired pneumonia is caused by a broad spectrum of microorganisms. Among these causative microorganisms, *S. pneumoniae* is able to produce numerous virulence factors capable of inducing significant host immune responses [11]. One of these factors, pneumolysin, has been proven to induce significant cytotoxicity in brain microvascular endothelial cells [12]. Such disruption in cerebrovascular endothelial function might have a detrimental impact on hemostatic balance and results in intravascular thrombosis [13].

The results of this study also support the use of measures to prevent pneumococcal infection in specific populations. The major measure for preventing pneumococcal diseases is vaccination with a polysaccharide pneumococcal vaccine. A marked reduction of myocardial infarction rate had been found after polysaccharide pneumococcal vaccination [14]. Surface protein-based pneumococcal vaccines might also be effective because they offer a broader spectrum protection against various serotypes [15]. We further conducted a study to explore whether the vaccination protect against pneumonia or stroke. In Taiwan, only individuals aged 75 or more could be vaccinated with pneumococcal vaccine, so this study consisted of individuals aged 75 or more between 2008 and 2009. Pneumococcal vaccine with influenza vaccine is associated with reduced risk of stroke and reduced pneumonia and influenza risk (Appendix S2). Pneumococcal vaccine alone is not statistically associated with reduced risk of stroke and pneumonia & influenza. Both influenza vaccine and pneumococcal vaccine may be suggested for the elderly with evidence-based results. Non-vaccination, post-infection measures might also be beneficial, including more intensive stroke prevention strategies. Given that 70% of the post-pneumococcal stroke events occurred within the first year after pneumococcal pneumonia and significantly increased risk of stroke during the first year, efforts to prevent vascular events should continue for a minimum of one year after the onset of pneumococcal pneumonia.

This study has several limitations. First, the diagnosis of pneumococcal pneumonia, appendectomy, stroke, and any other comorbid conditions are completely dependent on ICD codes. Nonetheless, the NHI Bureau of Taiwan has randomly reviewed the charts and interviewed patients to verify the diagnostic accuracy. Hospitals with outlier chargers or practices may undergo an audit, with subsequent heavy penalties for malpractice or discrepancies. We further conducted a sensitivity test under the positive predictive rate of 50% for ICD 481 [9]. Pneumococcal pneumonia was an independent risk factor for stroke during the first year of follow-up. Second, hospitalized patients with a principal diagnosis of pneumococcal pneumonia have been chosen to reduce misdiagnosis. However, the selection bias prevents generalization of the results to all pneumococcal pneumonia patients that may undergo treatment in the outpatient department. Third, detailed information on strokes, such as thrombotic and haemorrhagic stroke with different underlying pathogenesis mechanisms, cannot be extracted from ICD codes, which prevents further subgroup analysis. Fourth, the database does not contain information on tobacco use, dietary habits, and body mass index, which may also be risk factors for vascular events. However, we tried to simulate a randomization process in our observational study using instrumental variable analysis. IVA was designed to adjust potential biases associated with measured and unmeasured confounding variables, such as disease severity, tobacco use, dietary habits and social factors. Our instrumental variable is verified by comparing baseline characteristics and found these factors comparable.

In summary, this study is the first attempt to investigate the risk of cerebrovascular events in patients hospitalized for treatment of pneumococcal pneumonia. The results reveal that patients with pneumococcal pneumonia have increased risk of subsequent stroke and that their likelihood of developing stroke is increased during the first year. IVA showed an additional 14% risk of stroke for pneumococcal pneumonia patients. Influenza vaccine and pneumococcal vaccine may be suggested for the elderly. Prospective intervention studies of stroke prevention in the months after pneumococcal pneumonia are warranted.

## Supporting Information

Appendix S1 Adjusted hazard ratio of stroke among the pneumococcal pneumonia patients and the control group in the first year of follow-up under a positive predictive rate of 50% for pneumococcal pneumonia diagnosis in hospitalization records.
(DOC)

Appendix S2 Adjusted hazard ratios of stroke events and pneumococcal pneumonia & influenza among the individuals aged 75 or more with vaccination and those without vaccination.
(DOC)

## References

[1] Ross R (1999) Atherosclerosis–an inflammatory disease. N Engl J Med 340: 115–126. 9887164 10.1056/NEJM199901143400207

[2] Elkind MS, Luna JM, Moon YP, Boden-Albala B, Liu KM, et al (2010) Infectious burden and carotid plaque thickness: the northern Manhattan study. Stroke 41: e117–122. 20075350 10.1161/STROKEAHA.109.571299 PMC2830875

[3] Smieja M, Gnarpe J, Lonn E, Gnarpe H, Olsson G, et al (2003) Multiple infections and subsequent cardiovascular events in the Heart Outcomes Prevention Evaluation (HOPE) Study. Circulation 107: 251–257. 12538424 10.1161/01.cir.0000044940.65226.1f

[4] Libby P, Egan D, Skarlatos S (1997) Roles of infectious agents in atherosclerosis and restenosis: an assessment of the evidence and need for future research. Circulation 96: 4095–4103. 9403635 10.1161/01.cir.96.11.4095

[5] Mendall MA, Patel P, Asante M, Ballam L, Morris J, et al (1997) Relation of serum cytokine concentrations to cardiovascular risk factors and coronary heart disease. Heart 78: 273–277. 9391290 10.1136/hrt.78.3.273 PMC484930

[6] Smeeth L, Thomas SL, Hall AJ, Hubbard R, Farrington P, et al (2004) Risk of myocardial infarction and stroke after acute infection or vaccination. N Engl J Med 351: 2611–2618. 15602021 10.1056/NEJMoa041747

[7] Kellum JA, Kong L, Fink MP, Weissfeld LA, Yealy DM, et al (2007) Understanding the inflammatory cytokine response in pneumonia and sepsis: results of the Genetic and Inflammatory Markers of Sepsis (GenIMS) Study. Arch Intern Med 167: 1655–1663. 17698689 10.1001/archinte.167.15.1655 PMC4495652

[8] Ramirez J, Aliberti S, Mirsaeidi M, Peyrani P, Filardo G, et al (2008) Acute myocardial infarction in hospitalized patients with community-acquired pneumonia. Clin Infect Dis 47: 182–187. 18533841 10.1086/589246

[9] Guevara RE, Butler JC, Marston BJ, Plouffe JF, File TM, et al (1999) Accuracy of ICD-9-CM Codes in Detecting Community-acquired Pneumococcal Pneumonia for Incidence and Vaccine Efficacy Studies. American Journal of Epidemiology 149: 282–289. 9927225 10.1093/oxfordjournals.aje.a009804

[10] Angrist JD IG, Rubin DB (1996) Indentification of causal effects using instrumental variables. J Am Stat Assoc 91: 444–455.

[11] Kadioglu A, Weiser JN, Paton JC, Andrew PW (2008) The role of Streptococcus pneumoniae virulence factors in host respiratory colonization and disease. Nat Rev Microbiol 6: 288–301. 18340341 10.1038/nrmicro1871

[12] Zysk G, Schneider-Wald BK, Hwang JH, Bejo L, Kim KS, et al (2001) Pneumolysin is the main inducer of cytotoxicity to brain microvascular endothelial cells caused by Streptococcus pneumoniae. Infect Immun 69: 845–852. 11159977 10.1128/IAI.69.2.845-852.2001 PMC97961

[13] Gross PL, Aird WC (2000) The endothelium and thrombosis. Semin Thromb Hemost 26: 463–478. 11129402 10.1055/s-2000-13202

[14] Lamontagne F, Garant MP, Carvalho JC, Lanthier L, Smieja M, et al (2008) Pneumococcal vaccination and risk of myocardial infarction. CMAJ 179: 773–777. 18838452 10.1503/cmaj.070221 PMC2553874

[15] Tai SS (2006) Streptococcus pneumoniae protein vaccine candidates: properties, activities and animal studies. Crit Rev Microbiol 32: 139–153. 16893751 10.1080/10408410600822942

