# Supplementary material for: Pneumococcal Pneumonia and the Risk of Stroke: A Population-Based Follow-Up Study
Source: PLoS One. 2012 Dec 12;7(12):e51452. doi: 10.1371/journal.pone.0051452 (PMC3520842; doi:10.1371/journal.pone.0051452)
Supplement: Appendix S1 — Adjusted hazard ratio of stroke among the pneumococcal pneumonia patients and the control group in the first year of follow-up under a positive predictive rate of 50% for pneumococcal pneumonia diagnosis in hospitalization records. (DOC) [file pone.0051452.s001.doc]

Pneumococcal pneumonia group (n=745)

Control group

(n=1490)

Randomly select 373 patients as “real pneumococcal pneumonia group” (n=373)

372 patients

(n=372)

“Real control group”

(n=1862)

PPV=50% for ICD code

Regression analysis

*Appendix S1.

Procedure for sensitivity test:

1. Under the assumption of 50% positive predictive rate of ICD code for pneumococcal pneumonia, we randomly selected 50% of the pneumococcal pneumonia patients as “real pneumococcal pneumonia group”.
2. The other 372 patients from the pneumococcal pneumonia group was grouped with the control group (n=1490) as the “real control group”.
3. Then, Cox regression was performed.
4. We repeat the above mentioned procedures for 10 times.

| Appendix S1-continued.  Adjusted hazard ratio of stroke among the pneumococcal pneumonia patients and the control group in the first year of follow-up under a positive predictive rate of 50% for pneumococcal pneumonia diagnosis in hospitalization records. | | | | | |
| --- | --- | --- | --- | --- | --- |
| Variable* | | Event in the 1^st^ year | | | |
|  |  | No. of events | HR | 95% CI | P value |
| 1 | Real Pneumococcal pneumonia group (n=373) | 31 | 2.26 | (1.41-3.62) | 0.001 |
|  | Real Control group (n=1862) | 54 | 1 |  |  |
| 2 | Real Pneumococcal pneumonia group (n=373) | 27 | 2.14 | (1.34-3.43) | 0.001 |
|  | Real Control group (n=1862) | 58 | 1 |  |  |
| 3 | Real Pneumococcal pneumonia group (n=373) | 29 | 2.20 | (1.38-3.50) | 0.001 |
|  | Real Control group (n=1862) | 56 | 1 |  |  |
| 4 | Real Pneumococcal pneumonia group (n=373) | 28 | 2.17 | (1.35-3.47) | 0.001 |
|  | Real Control group (n=1862) | 57 | 1 |  |  |
| 5 | Real Pneumococcal pneumonia group (n=373) | 30 | 2.49 | (1.56-3.98) | 0.001 |
|  | Real Control group (n=1862) | 55 | 1 |  |  |
| 6 | Real Pneumococcal pneumonia group (n=373) | 34 | 3.14 | (2.00-4.94) | <0.001 |
|  | Real Control group (n=1862) | 51 | 1 |  |  |
| 7 | Real Pneumococcal pneumonia group (n=373) | 26 | 1.73 | (1.05-2.84) | 0.031 |
|  | Real Control group (n=1862) | 59 | 1 |  |  |
| 8 | Real Pneumococcal pneumonia group (n=373) | 28 | 2.02 | (1.26-3.26) | 0.004 |
|  | Real Control group (n=1862) | 57 | 1 |  |  |
| 9 | Real Pneumococcal pneumonia group (n=373) | 29 | 2.19 | (1.37-3.51) | 0.001 |
|  | Real Control group (n=1862) | 56 | 1 |  |  |
| 10 | Real Pneumococcal pneumonia group (n=373) | 28 | 2.14 | (1.34-3.41) | 0.002 |
|  | Real Control group (n=1862) | 57 | 1 |  |  |
| Abbreviation: 95%CI, 95% confidence interval; HR, hazard ratio. | | | | | |

*10 trials of sensitivity test.
