# Supplementary material for: Pneumococcal Pneumonia and the Risk of Stroke: A Population-Based Follow-Up Study
Source: PLoS One. 2012 Dec 12;7(12):e51452. doi: 10.1371/journal.pone.0051452 (PMC3520842; doi:10.1371/journal.pone.0051452)
Supplement: Appendix S2 — Adjusted hazard ratios of stroke events and pneumococcal pneumonia & influenza among the individuals aged 75 or more with vaccination and those without vaccination. (DOC) [file pone.0051452.s002.doc]

| Appendix S2. Adjusted hazard ratios of stroke events and pneumococcal pneumonia & influenza among the individuals aged 75 or more with vaccination and those without vaccination* (n=35300) | | | | | | | |
| --- | --- | --- | --- | --- | --- | --- | --- |
|  | Stroke event | | |  | Pneumonia and influenza | | |
| Variable | No. of stroke events | Multivariate adjusted HR (95% CI) | P |  | No. of pneumonia and influenza | Multivariate adjusted HR (95% CI) | P |
| Without vaccine (n=17149) | 913(5.3) | 1 |  |  | 346(2.0) | 1 |  |
| Influenza vaccine (n=10229) | 495(4.8) | 0.88(0.79-0.99) | 0.028 |  | 186(1.8) | 0.86(0.72-1.03) | 0.091 |
| Influenza vaccine and pneumococcal vaccine (n=7561) | 295(3.9) | 0.72(0.63-0.83) | <0.001 |  | 93(1.2) | 0.61(0.48-0.77) | <0.001 |
| Pneumococcal vaccine (n=361) | 11(3.0) | 0.57(0.31-1.03) | 0.061 |  | 3(0.8) | 0.42(0.14-1.32) | 0.139 |
| CI, confidence interval; HR, hazard ratio.  *Adjusted for age, gender, OPD amount, diabetes, chronic lung disease, chronic kindey disease, chronic heart disease, immunodeficiency, cancer, geographic area and urbanization of residence, and socioeconomic status. | | | | | | | |
